# The Impact of Volunteering and Its Characteristics on Well-being After State Pension Age: Longitudinal Evidence From the English Longitudinal Study of Ageing

**DOI:** 10.1093/geronb/gbaa146

**Published:** 2020-09-05

**Authors:** Katey Matthews, James Nazroo

**Affiliations:** CMIST, University of Manchester

**Keywords:** Activity theory, Ageing, Mental well-being, Retirement, Role theory

## Abstract

**Objectives:**

Volunteering after retirement age is beneficial to well-being. This study furthers previous research by presenting a longitudinal analysis of the well-being of volunteers, compared to non-volunteers, based on characteristics of the voluntary work in which they participate.

**Method:**

Participants were 3,740 people aged State Pension Age and over from the English Longitudinal Study of Ageing. Longitudinal regression models were used to determine whether frequent volunteers saw beneficial changes in well-being (depression, satisfaction with life, CASP-19, and social isolation) compared to non-volunteers. The initial model used a hierarchical approach so that we could also examine the impact of social and health factors. Models were then run to determine whether well-being in relation to volunteering was influenced by its continuity, the number of activities engaged in, whether the volunteering was formal or informal in nature, and whether or not the respondent reported feeling appreciated for their efforts.

**Results:**

Although sociodemographic and health circumstances reduce the magnitude of the effects of volunteering on well-being, the effect of volunteering remained significant in almost all analyses. The beneficial effect of volunteering appeared to stop among respondents who stopped volunteering between waves. The best outcomes were observed among those participating in higher numbers of activities, regardless of whether or not these were classed as formal or informal, and who felt appreciated for their work.

**Discussion:**

Certain aspects of volunteering might be especially beneficial to the well-being of older people. That these effects stop when volunteering stops suggest a causal element to this relationship.

Rather than defining retirement as an inevitable withdrawal from society, loss of productive activity, and cause of financial instability (Moen, 1996), contemporary theory draws on the idea that retirement enables engagement in socially productive and meaningful activity, leading to better social and mental well-being (Bound & Waidmann, 2007), and that continued meaningful social engagement is a crucial component of healthy ageing (Greenfield & Marks, 2007). One way of continuing social activity in later-life is voluntary work. This study provides a longitudinal analysis of the effects of volunteering post-State Pension Age (SPA), that is, the age at which one is eligible to receive their government pension benefit, on well-being, considering volunteering as a multidimensional concept in terms of its characteristics.

Much research has focused on volunteering in later life, suggesting volunteering may be of particular benefit to older individuals (Hao, 2008; Li & Ferraro, 2006; Musick & Wilson, 2003; Tabassum et al., 2016). Indeed, the value of volunteering may change across the lifecourse, with a potential stronger impact on well-being for those who are retired. This is perhaps because volunteering can be a substitute for previously held roles (Greenfield & Marks, 2007; Li & Ferraro, 2006; Wahrendorf et al., 2008), and because those who volunteer post-retirement do so without the pressure of simultaneous employment (Greenfield & Marks, 2004). Substituting employment with volunteering also ensures individuals remain integrated in society, maintaining social interaction that, in turn, enables better access to social engagement and support, resources, and contacts (Hao, 2008; Mackenzie & Abdulrazaq, 2019; Mellor et al., 2009; Morrow-Howell et al., 2003; Musick & Wilson, 2003). Volunteering may also counteract feelings of loss of power and social status among individuals no longer working (Greenfield & Marks, 2004; Musick et al., 1999). Furthermore, the altruistic nature of volunteering may improve individual self-esteem over and above these other effects (Li & Ferraro, 2006; Thoits & Hewitt, 2001; Yeung et al., 2018).

There is a large body of evidence demonstrating volunteering’s relationship with better health and social outcomes (Jenkinson et al. 2013). More specifically, research has shown participation in voluntary work is associated with: higher self-esteem (Greenfield & Marks, 2004), improved self-efficacy, and lower negative affect (Musick et al., 1999); reduced depression (Chiao et al., 2011; Kim & Pai, 2010; Lawrence et al., 2019; Matthieu et al., 2017; Musick & Wilson, 2003; Musick et al., 1999); better quality of life (Cattan et al., 2011); and higher life satisfaction (Haski Levanthal, 2009). Studies also show associations between volunteering and physical health, including functioning (Lum & Lightfoot, 2005), mortality (Harris & Thoresen, 2005; Musick et al., 1999; Rogers, Demakakos, et al., 2016), and hypertension (Sneed & Cohen, 2013).

Evidence has shown time spent volunteering, as well as the number of volunteering activities engaged in may be linked with individual well-being, with a “midpoint” of optimum engagement providing the best outcomes on well-being (Greenfield & Marks, 2007; Jirovec and Hyduk, 1999; Morrow-Howell et al., 2003). Research by Windsor et al. (2008) specified this point to be around 15 hr per week, a level substantial enough to protect from the negative effects of workforce withdrawal on the individual’s perception of their social productivity, meaningfulness, and membership of social networks, while simultaneously being manageable enough to not be a burden and to enable enjoyment of the freedom from constraints of working life (Musick et al., 1999; Thoits, 2012; Windsor et al., 2008). Mackenzie and Abdulrazaq (2019) further demonstrate higher participation in social activities, such as volunteering, was associated with poorer mental well-being after controlling for social engagement, suggesting the level of participation in such activities should be just enough to secure a level of social engagement beneficial to the individual.

The extent to which individuals feel appreciated for volunteering may also determine the strength of its impact on well-being. Research shows individuals working in jobs with a good effort–reward balance have significantly better mental health (Godin et al., 2005; Niedhammer et al., 2006; Siegrist, 1996; Zaninotto et al., 2013), self-reported health (Niedhammer et al., 2004), cardiovascular health and mortality (Siegrist, 2010). Given that a key factor in the beneficial effect of volunteering is argued to be its ability to replace employment-type roles following retirement, it is important to consider whether these effects will be greatest when the voluntary work carried out is reciprocated, as in the workplace.

Hoereth (2010) defines formal volunteering as that which occurs within an organizational setting, providing well-defined roles and the potential to cover expenses. More informal volunteering is, conversely, likely to be less well structured and focused more on personal help and care, sometimes leading to greater intensity of work and less-evident outcomes. Evidence suggests more formal types of volunteering, such as raising money and campaigning, are associated with better well-being outcomes (Greenfield & Marks, 2004; Mutchler et al., 2003), perhaps because it is more likely to mimic tasks carried out as part of working life, contributing to social inclusion, value, and purpose, as well as maintaining networks and resources that otherwise may be lost through retirement (Greenfield & Marks, 2004; Musick et al., 1999; Thoits, 2012). However, while less formal types of volunteering may veer away from workforce type settings, they may be more altruistic in nature and therefore provide the individual with feelings of higher self-esteem and positive affect, and consequent better well-being (Yeung et al. 2018).

This study expands on previous research in two key ways. First, we stratify volunteering to examine whether the magnitude of its effects are dependent on its characteristics. Second, we use a multidimensional concept of mental health and well-being (Vanhoutte & Nazroo, 2014), in order to understand volunteering in relation to the individual’s overall life quality (Mellor et al., 2009). The paper focuses on retired individuals of SPA or older, examining longitudinal associations between volunteering and changes in well-being over time. The study explicitly explores the changes in well-being of volunteers compared to non-volunteers. We hypothesize volunteers see positive changes in well-being compared with non-volunteers and, furthermore, those effects will be of a larger magnitude when the volunteering is well reciprocated, formal in nature, and when a greater amount of time is invested in voluntary activities.

## Method

Data are used from the English Longitudinal Study of Ageing (ELSA) (Rogers, Banks, et al., 2016; Steptoe et al., 2013), a multidisciplinary survey of a nationally representative sample of people aged 50 and over living in England. Data are collected biennially, with Wave 1 collected in 2002 and the latest wave (Wave 8) collected in 2016–2017.

### Sample

This study uses Waves 7 and 8 of ELSA (2016–2018). Inclusion of earlier waves would result in fewer participants due to survey drop out and discontinuation of volunteering over time. Additionally, more complex missing data procedures would lead to potential reductions in statistical efficiency and would only partially address non-response biases. The sample includes respondents of SPA and over at the time of data collection (for men, age 65 and women, age 63).The analysis includes only those who answered relevant questions at Waves 7 and 8 of the data, giving a total of 3,740 individuals.

### Measures

#### Volunteering

ELSA respondents were asked how often they participated in voluntary work, with the options *twice a month or more, about once a month, every few months, about once or twice a year, less than once a year and never*. Respondents were included in the study if they answered at least once a month. Individuals who reported never volunteering formed the reference group of non-volunteers. Those who volunteered less than once a month are excluded from the analysis. This definition of volunteering is in line with similar research (Haski Levanthal, 2009; Rogers, Demakakos, et al. 2016; Zaninotto et al., 2013).

#### Reciprocity of voluntary work

As referenced earlier, much work has demonstrated strong links between various dimensions of effort and reward and the well-being of workers. ELSA respondents were asked “do you feel adequately appreciated for your voluntary work?” with the response options of strongly agree, agree, disagree, and strongly disagree. These were reduced to two categories, combining those who answered strongly agree and agree and those who answered strongly disagree and disagree. Feeling obliged to volunteer was already coded as a binary “yes” or “no” variable. Respondents were classed as being reciprocated for their volunteering if they agreed they were and also did not feel obliged to carry out their work.

#### Formal and informal volunteering

Respondents are asked whether or not they have taken part in a list of 13 specific formal volunteering activities (raising money, leading a group or committee, organizing activities or events, visiting, befriending or mentoring, teaching, counseling, secretarial work, providing transport, representing, campaigning, other practical help or other help within an organizational setting) and 8 informal activities focusing on more personal unpaid tasks (helping with household tasks, decorating, babysitting, providing personal care, looking after a property or pet, writing letters, representing someone and transporting or escorting someone). A continuous score of the sum of formal and informal tasks is generated for those who volunteer (range 1–19), and is then categorized to show those participating in one, two, or three or more activities.

#### Well-being

This study focuses on four key domains of mental health and well-being previously demonstrated to be significantly associated with volunteering. First, depressive symptomatology was measured using an abbreviated eight-item version of the Center for Epidemiological Studies-Depression scale (CES-D) (Radloff, 1977), with scores ranging from 0, indicating no symptoms of depression, to 8. Second, quality of life was measured using a 16-item version of the CASP-19 score (Vanhoutte & Nazroo, 2014), which is a scale derived from 19 questions relating to control, autonomy, self-realization, and pleasure (CASP) in later life. Scores range from 0 to 57, with higher scores indicating better quality of life. Third, life satisfaction was measured using the Diener et al. (1985) five-item scale. Scores range from 1 to 35, with higher scores indicating better life satisfaction. All three of these scales have been shown to have high validity and reliability within studies of ageing populations (Vanhoutte & Nazroo, 2014). Fourth, social isolation was measured using a modified version of the UCLA Loneliness Scale, a five-item questionnaire reflecting lack of companionship, feeling left out, feeling isolated, feeling out of tune with others, and feeling lonely (Hughes et al., 2004). Here, scores range from 1 to 15, with higher scores indicating higher isolation. The current study will expand on previous work examining multiple dimensions of mental well-being in order to investigate whether outcomes are affected differently by the various characteristics of voluntary work.

#### Demographic, socioeconomic, and physical well-being measures

The analyses control for potentially confounding individual characteristics. All analyses adjust for sex, and age as a continuous variable. Marital status is included as a binary variable, differentiating between those who are married or cohabiting and those who are single, widowed, or divorced. Wealth is measured in terms of net total non-pension wealth at the household level and is included as a quintile variable. Wealth is included over income, social class, or education as it is argued to better reflect older peoples’ cumulative social status (de Oliveira et al. 2010). Self-reported health is included as a categorical variable with responses excellent, very good, good, fair, and poor. Analyses also control for participation in paid employment and provision of care for the elderly or sick by means of two binary variables.

### Statistical Analysis

The longitudinal models use Waves 7 (*t*-1) and 8 (*t*) of the ELSA data to examine the impact of volunteering on well-being using linear regression models adjusted for baseline well-being, denoted as:

$$\begin{array}{ll} Well\text{-}being_{t} & =Well\text{-}being_{t-1}+Volunteering\;status_{t-1}\;\\ & +Controls_{t-1}\;+\;Constant \end{array}$$

Coefficients reported indicate both the size and direction of relative change between waves in well-being scores for those who volunteer compared with those who do not. In the initial model, a hierarchical approach is taken to adding controls, so that their contribution to the basic associations between volunteering and well-being can be examined.

To examine whether the relationship between volunteering and well-being scores is dependent on the consistency of volunteering, the second analysis compares the well-being of those who volunteer at both Waves 7 and 8 with those who, firstly, volunteer only at Wave 7 and who have stopped by Wave 8 and, secondly, do not volunteer at Wave 7 but do at Wave 8. Thirdly, a comparison is drawn between changes in well-being of volunteers relative to non-volunteers on the basis of the number of activities they participate in. The fourth model examines whether there is an impact on well-being dependent on whether volunteering is in formal or informal domains, and whether this relationship is dependent on the number of formal or informal activities carried out. Fifth, whether the well-being of volunteers, relative to non-volunteers, is better when they feel the voluntary work they carry out is appreciated is examined.

The analyses of changes over time use the Wave 8 longitudinal weights, while descriptive analyses of the characteristics of volunteers use the Wave 7 cross-sectional weights, both to deal with issues arising from attrition. Analyses were carried out using Stata 14.

## Results

### Characteristics of Volunteers and Non-Volunteers

Table 1 shows information on the characteristics of volunteers, controlled for as potential confounders in the further models, compared to non-volunteers at baseline, and then, for volunteers, the characteristics of the voluntary work they carry out. With the exception of participation in paid work, all group differences are significant (*p* < .001).

**Table 1.** Characteristics of Volunteers and Non-Volunteers (a) and the Voluntary Work of Those Who Do Volunteer (b) (Baseline [Wave 7])

	Volunteering status at baseline (Wave 7)	
	Volunteer (%)	Non-volunteer (%)
(a) Characteristics of volunteers and non-volunteers		
Female	59.43	57.83
Age		
Male mean age	72.35	74.47
Female mean age	71.35	74.07
Ethnicity		
Non-white	2.67	3.16
Marital status		
Married or cohabiting	71.37	62.93
Wealth		
Poorest	9.53	20.40
2nd	12.35	19.07
Middle	21.23	22.56
4th	25.99	20.75
Wealthiest	30.91	17.22
Self-reported health		
Excellent	12.50	6.74
Very good	35.68	24.75
Good	34.40	33.1
Fair	14.39	24.96
Poor	3.03	10.44
Participation in other activities		
In paid work	8.49	9.14
Provides care	18.18	9.56
(b) Of those who volunteer		
Does not feel appreciated for volunteering	8.23	
Volunteers at Waves 7 and 8	59.29	
Stops volunteering by Wave 8	22.71	
Starts volunteering by Wave 8	18.00	
Participates in three or more activities	71.16	
Participates in formal volunteering	90.64	
One activity	19.72	
Two or more activities	80.28	
Participates in informal volunteering	56.97	
One activity	49.89	
Two or more activities	50.11	
Unweighted base	1,127	2,613

The mean age of volunteers is similar among both males and females, with volunteers around 2 and 3 years younger than non-volunteers, respectively. There is a slightly higher proportion of non-white individuals not volunteering than volunteering. A higher proportion of volunteers than non-volunteers are married or cohabiting. There is a strong linear association between volunteering and wealth, with the percentage of volunteers increasing as wealth quintile increases. Non-volunteers are more evenly distributed over the five wealth quintiles, although half as many non-volunteers than volunteers belong to the wealthiest quintile, and over twice as many belong to the poorest. A much higher percentage of non-volunteers than volunteers report poor health, and the proportion of volunteers reporting excellent health is twice that of non-volunteers. Finally, volunteers are more likely to participate in the provision of care than non-volunteers.

Approximately, 30% of people over SPA volunteer frequently. Of volunteers, the vast majority feel appreciated for the voluntary work they carry out. Around 60% of respondents volunteer at both waves of the data. When considering any type of voluntary work (formal or informal), around 70% of volunteers engage in at least three different volunteering activities. If we break those activities down by their type, around 90% participate in “formal” activities, and four fifths of those participate in two or more formal activities. In contrast, just over half of volunteers report engaging in “informal” volunteering activities, and furthermore, only half of informal volunteers engage in more than one informal activity.

### Change in Well-being for Volunteers Compared With Non-Volunteers

The results of the longitudinal models investigating change in well-being on the basis of participation in voluntary work are shown in Table 2. All models adjust for baseline well-being to demonstrate relative change in well-being over the 2-year data period. Change is measured for those who volunteer compared to those who do not. For depression and social isolation, improvement is indicated by a negative coefficient, while for quality of life and life satisfaction improvements are indicated by positive coefficients.

**Table 2.** Change in Well-being Over 2 Years: Volunteers Compared With Non-Volunteers (Results From Hierarchical Regression: Regression Coefficients and *SE*s)

	Baseline score	+ Age, sex, and marital	+ Wealth	+ Self-reported health	+ Paid work and caring
Depression	0.293 (0.06)***	0.290 (0.06)***	0.219 (0.06)***	0.190 (0.06)***	0.189 (0.06)***
Life satisfaction	1.019 (0.17)***	1.011 (0.17)***	0.916 (0.18)***	0.832 (0.17)***	0.829 (0.17)***
Quality of life	1.112 (0.20)***	1.022 (0.20)***	0.891 (0.21)***	0.718 (0.21)**	0.703 (0.21)**
Social isolation	0.162 (0.06)*	0.145 (0.06)*	0.121 (0.07)	0.080 (0.07)	0.084 (0.07)

*Notes*: Columns adjusted as follows: column 1, baseline well-being; column 2 + age, sex, and marital status; column 3 + wealth quintile; column 4 + self-reported health; column 5 + paid work and caregiving.

*p < .05. **p < .01. ***p < .001.

The first column in Table 2 shows the basic associations between volunteering and well-being, adjusting only for baseline outcomes. Coefficients represent the relative change in well-being for volunteers compared to non-volunteers, so, for example, the decrease in depression score of −0.293 represents the change in depression score for volunteers compared to the change in depression score for non-volunteers.

In Model 1, which includes only the baseline well-being score, well-being significantly improves across all outcomes for volunteers compared to non-volunteers. In each instance, these associations remain significant after adjusting for demographics. After controlling for wealth, all outcomes except social isolation remain significant, although coefficients become smaller. Although controlling for self-reported health and participation in other activities leads to a decrease in the magnitude of the coefficients, the life satisfaction and quality of life of volunteers remain significantly better than that of non-volunteers, and levels of depression remain significantly lower.

### Consistency and Amount of Volunteering and Change in Well-being

Table 3 shows the results of models testing whether beneficial associations between volunteering and well-being persisted for individuals who volunteered but then stopped. Here, the change in well-being score between Waves 7 and 8 for those who reported volunteering at both waves relative to those who did not volunteer at all, is compared to the change in well-being reported by those who volunteered at Wave 7 or Wave 8 only. Table 3 also shows whether effects of volunteering are dependent on the number of activities undertaken.

**Table 3.** Change in Well-being Over 2 Years and Continuity and Amount of Volunteering: Volunteers Compared With Non-Volunteers (Regression Coefficients and *SE*s)

	Continuity of volunteering			Number of volunteering activities participated in	
	Volunteers at Waves 7 and 8	Volunteers at Wave 7 only	Volunteers at Wave 8 only	One or two	Three or more
Depression	−0.182 (0.06)**	0.176 (0.11)	−0.173 (0.08)*	−0.146 (0.09)	−0.225 (0.06)***
Life satisfaction	0.769 (0.21)***	−0.121 (0.37)	0.633 (0.22)**	0.011 (0.30)	0.491 (0.22)*
Quality of life	1.249 (0.23)***	−0.087 (0.41)	1.177 (0.43)***	0.815 (0.38)*	0.741 (0.22)**
Social isolation	−0.150 (0.07)*	0.139 (0.14)	−0.177 (0.14)	−0.087 (0.011)	−0.111 (0.07)

*Notes*: Results of fully adjusted models shown only.

*p < .05. **p < .01. ***p < .001.

The results in Table 3 suggest there are differences in the effects of volunteering continuously, stopping volunteering and taking up volunteering in comparison with respondents who do not volunteer at all. In models adjusting for baseline well-being only, those who volunteered at both Waves 7 and 8 or who started volunteering between Waves 7 and 8 saw large and significant improvements in all areas of well-being compared to those who did not volunteer at either wave. The well-being of those who stopped volunteering saw better improvements than those who did not volunteer at all, but these effects are not significant. Results remained significant in the fully adjusted models except in the instance of social isolation, although coefficients reduced in size. Considering the number of volunteering activities individuals engage in, a larger beneficial effect is observed on well-being of participation in at least three or more activities rather than one or two, and this result is again significant for all outcomes except social isolation.

### Type of Volunteering and Change in Well-being

The set of results presented in Table 4 focuses on the associations between the type and, within each type, the amount of volunteering and well-being. Again, the change in well-being is examined for those who volunteer compared to those who do not.

**Table 4.** Change in Well-being Over 2 Years and Type and Amount of Volunteering: Volunteers Compared With Non-Volunteers (Regression Coefficients and *SE*s)

	Depression	Life satisfaction	Quality of life	Social isolation
Formal vs informal volunteering^a^				
Formal	−0.173 (0.08)*	0.489 (0.23)*	0.699 (0.29)*	−0.020 (0.10)
Informal	−0.111 (0.09)	0.181 (0.22)	0.102 (0.32)	−0.082 (0.11)
Formal volunteering				
One activity	−0.014 (0.13)	0.050 (0.42)	0.563 (0.40)	−0.026 (0.13)
Two+ activities	−0.249 (0.06)***	0.458 (0.21)*	0.756 (0.21)***	−0.081 (0.07)
Informal volunteering				
One activity	−0.255 (0.09)**	0.220 (0.30)	0.191 (0.34)	−0.074 (0.11)
Two+ activities	−0.219 (0.08)**	0.887 (0.32)**	0.809 (0.27)**	−0.227 (0.08)**

*Notes*: ^a^Fully controlled models additionally control for number of activities engaged in.

*p < .05. **p < .01. ***p < .001.

In relation to formal and informal volunteering, significant relationships are found only for depression and quality of life, where respondents in formal volunteering observe better changes to their well-being than those in informal positions, relative to non-volunteers. The magnitude of the effects on well-being of participating in two or more activities is larger than those for individuals participating in just one activity. The effects of participating in two or more formal activities remain significant in the fully adjusted models in each instance, except the decrease in social isolation, and always show a beneficial association with well-being. Participation in just one formal activity is only associated with improved quality of life compared to no volunteering activity at all, although this result becomes nonsignificant after adjusting for all other factors. Participation in at least two informal volunteering activities is also associated with significantly better well-being than no volunteering for each of the outcomes in both the unadjusted and adjusted models. Additionally, participation in just one informal activity shows a significant reduction in depression scores compared to those who do not volunteer at all.

### Voluntary Work, Reciprocity, and Change in Well-being

Table 5 shows whether or not feeling well reciprocated for participating in voluntary work is associated with the well-being of volunteers. Again, change in well-being is measured over a 2-year period for those who felt reciprocated for their participation in volunteering and those who did not, both relative to non-volunteers. Results are presented only for the final model, adjusting for all covariates (the equivalent to column 5 in Table 2).

**Table 5.** Change in Well-being Over 2 Years and Feeling Appreciated for Volunteering: Volunteers Compared With Non-Volunteers (Regression Coefficients and *SE*s)

	Appreciated	Unappreciated
Depression	−0.199 (0.06)***	−0.028 (0.19)
Life satisfaction	0.419 (0.20)*	−0.248 (0.62)
Quality of life	0.773 (0.21)***	0.130 (0.51)
Social isolation	−0.093 (0.07)	−0.026 (0.15)

*Notes*: Results of fully adjusted models shown only.

*p < .05. ***p < .001.

Table 5 suggests that the well-being of volunteers who feel appreciated for their voluntary work improves significantly more than the well-being of non-volunteers over a two year period. When controlling for only baseline well-being, well-reciprocated volunteering is associated with significantly better well-being across all outcomes when compared to the well-being of those who do not volunteer. However, only the effects on depression, life satisfaction, and quality of life remain significant in the model adjusting for demographic factors, wealth, health, and participation in other roles. The effects of unappreciated volunteering relative to no volunteering on well-being are much smaller and are not statistically significant in either the unadjusted or adjusted models.

## Discussion

This study set out to examine the longitudinal relationship between various characteristics of volunteering and change in well-being for adults over SPA in England. Around 30% of ELSA respondents reported volunteering more than once a month. Descriptive analysis showed volunteers were likely to be wealthier and healthier than non-volunteers. Examining change in outcomes over a 2-year period demonstrated the well-being of volunteers improved relative to that of non-volunteers. Although the size of the improvement reduced after adjusting for social and economic differences between volunteers and non-volunteers, it remained significant for outcomes of depression, satisfaction with life, and quality of life. The longitudinal nature of this analysis and adjustment for social and economic factors suggests evidence of a causal relationship between volunteering and well-being in later life. This conclusion is strengthened by the finding that higher numbers of volunteering activities are associated with larger improvements in well-being and improvements in volunteers’ well-being do not remain for those who stop volunteering between waves. Additionally, improvement in well-being is only observed among those who feel appreciated for the work they do.

This research ties in well with previous evidence on effects of volunteering on well-being and the mechanisms through which these might operate. The majority of our sample do not participate in paid employment (92%) and in line with activity and role theories, we hypothesized older people who volunteered would demonstrate better well-being than those who did not, due to continued participation in socially meaningful roles after retirement.

We drew on Siegrist’s (1996) model of effort–reward imbalance, which demonstrates strong associations between activity, reciprocity, and well-being (Godin et al., 2005; Niedhammer et al., 2006, 2004; Siegrist, 1996, 2010). In line with prior research demonstrating the beneficial effects of later-life working are only present in favorable conditions (Godin et al., 2005; Niedhammer et al., 2006, 2004; Siegrist, 1996, 2010), this study suggests volunteering is also only beneficial if the conditions of the voluntary work are positive.

In line with role accumulation theory (Moen et al., 1992), individuals participating in more volunteering observed better well-being. Fully adjusted models showed significantly better life satisfaction, quality of life, and depression among individuals who participated in three or more activities. Our findings again complement activity and role theories of ageing: that greater levels of volunteering “mimic” employment after retirement, allowing a continuation of the benefits to well-being of social engagement and involvement in socially meaningful exchanges (Thoits, 2012). However, when considering the “midpoint” of engagement, ELSA does not contain many respondents reporting more than three activities, or details of the time spent on activities, so the study was unable to reliably establish whether even higher numbers of activities would be detrimental to well-being (Mackenzie & Abdulrazaq, 2019; Morrow-Howell et al., 2014).

Formal, as opposed to informal, volunteering showed significant beneficial effects in terms of depression and quality of life. In terms of informal volunteering, changes in all outcomes were significantly better among those participating in two or more activities. This is at odds with some previous work which has shown informal tasks, particularly at higher levels, can lead to poorer well-being (Mutchler et al., 2003) through a lack of appreciation and reward and a higher risk of burnout (Li & Ferraro, 2005). However, our finding is in line with research demonstrating informal volunteering as altruistic and associated with better well-being through mechanisms of increased self-esteem (Yeung et al., 2018). That social isolation was only significantly reduced by at least two informal volunteering activities also lends credence to this theory.

There were no significant associations between volunteering and social isolation. Although included in the study as a well-being outcome, social isolation may instead be reflecting fewer social resources, strongly associated with wealth and health, and acting as a contributor to well-being, rather than as a dimension itself (Niedzwiedz et al., 2016).

The present study has several strengths. ELSA is a large nationally representative dataset, allowing longitudinal analysis of many people over SPA. The dataset provides a rich set of information on individuals’ volunteering characteristics that can be monitored over time in order to establish causality. ELSA also has detailed information on individual well-being, allowing examination of outcomes specifically linked to volunteering in later life. Furthermore, these measures are often tailored for older populations, such as the theoretically grounded CASP score and measures of social isolation.

There are some limitations to the work presented here to be considered. As with any longitudinal dataset, ELSA is affected by sample attrition. Analysis of attrition in ELSA demonstrates those most likely to drop out are older and in unfavorable socioeconomic and health circumstances (Steptoe et al. 2013). Furthermore, descriptive statistics showed volunteers to be healthier and wealthier than non-volunteers, potentially biasing positive outcomes on mental well-being. However, sample weights were used in order for results to be considered representative of the general English population over SPA.

Previous research into well-being and volunteering found selection into volunteering occurred simultaneously with the effects of volunteering (Li & Ferraro, 2005; Rogers, Demakakos, et al., 2016; Thoits & Hewitt, 2001). To address this, the study uses the longitudinal nature of the data in an attempt to establish an estimate of causality, and models also control for baseline socioeconomic and health factors. The analysis of the effect of continued, versus discontinued, volunteering, demonstrating the impact of volunteering only remained significant when it was continuous, and the demonstration of effects in relation to both quantity and frequency of volunteering, additionally lends credence to the causal nature of the analysis. However, the focus on changes in well-being will not include causal effects that have occurred at or before the initial time point of the study, and, as a result, estimates of the impact of volunteering on well-being may be conservative.

As ELSA is not primarily a study of volunteering, some measures may be biased by self-reporting, including the measure of volunteering itself which is restricted to a set of broad categories of frequency rather than something more detailed, such as hours spent volunteering. Furthermore, although the data provide a range of characteristics of voluntary work, some measures lack detail which might otherwise allow for a more elaborate analysis, for example, information on whether or not “reciprocity” was felt emotionally or financially. Research has shown reciprocity shares strong ties with the formality of volunteering (Yeung et al., 2018), but again, it was not possible to disentangle such effects using the current data. Information on the characteristics of the voluntary work of those who report volunteering less frequently, and were therefore omitted from the category of volunteers, might have instead made for a meaningful analysis of their efforts. Individuals volunteering infrequently might be focusing on much more intense tasks than those volunteering on a weekly basis, yet the data provide no means of assessing this. Attempts at establishing causal effects by means of examining discontinuation of volunteering might be more informative with the availability of details of how, why, and when individuals stopped volunteering, for example, whether they stopped all volunteering activities at once due to poor health or slowly over time due to participation in other social activities.

Finally, differences on the volunteering experience by gender are well documented (Moen et al., 1992). Although all models control for sex, sample sizes would have been drastically reduced by examining some of the characteristics of volunteering by gender individually.

Important policy implications can be taken from this study. The findings suggest clear associations between volunteering and well-being after retirement age. These findings fit alongside the results of other research demonstrating links specifically between older age and beneficial effects on well-being of volunteering (Lawrence et al., 2019; Matthieu et al., 2017; Rogers, Demakakos, et al., 2016; Zaninotto et al., 2013). As selection into volunteering is strongly affected by older age and declining physical health (Li & Ferraro, 2006; Papa et al., 2019; Thoits & Hewitt, 2001), increases to SPA puts older people at risk of being unable to participate in voluntary work after retirement, in turn placing the individual at risk of a loss of social involvement and status which is crucial to their well-being. It should also be noted that while individuals in poorer physical health might be less likely to volunteer, evidence still suggests that even a low level of engagement is better for well-being than none (Morrow-Howell et al., 2003), and so volunteering opportunities should be made available for all capabilities where possible. Similarly, previous work has demonstrated selectivity into volunteering on the basis of education and socioeconomic factors (Herzog & Morgan, 1993), yet research has also demonstrated strong beneficial effects of volunteering on well-being among these groups within society (Fried et al., 2013; Lawrence et al., 2019; Matthieu et al., 2017; Yeung et al., 2018). Therefore, policy should focus on providing volunteering opportunities for more disadvantaged socioeconomic groups, but with attention paid to the quality of those volunteering opportunities.
